# Research on the Applications of Calcium Propionate in Dairy Cows: A Review

**DOI:** 10.3390/ani10081336

**Published:** 2020-08-03

**Authors:** Fan Zhang, Xuemei Nan, Hui Wang, Yuming Guo, Benhai Xiong

**Affiliations:** 1 State Key Laboratory of Animal Nutrition, Institute of Animal Science, Chinese Academy of Agricultural Sciences, Beijing 100193, China; 18813015831@139.com (F.Z.); xuemeinan@126.com (X.N.); wanghui_lunwen@163.com (H.W.); 2 State Key Laboratory of Animal Nutrition, College of Animal Science and Technology, China Agricultural University, Beijing 100193, China; guoyum@cau.edu.cn

**Keywords:** calcium propionate, dairy cow, perinatal period, NEB, mycotoxin

## Abstract

**Simple Summary:**

In modern dairy cattle production systems, the mycotoxins in feed and metabolic disease, such as ketosis and milk fever, seriously affect the health and milk production of dairy cows. Calcium propionate is a safe and reliable food and additive that is widely used. It can be employed in silage and total mixed rations (TMR) against mycotoxin production. In the perinatal period, many cows cannot adjust to the tremendous metabolic, endocrine, and physiological changes, resulting in ketosis and fatty liver due to a negative energy balance or milk fever induced by hypocalcemia, which damages their health and reduces the production performance. Studies have revealed that calcium propionate can play an active role in solving these problems. It can also regulate rumen development in calves. This paper reviews the recent research progress regarding the application of calcium propionate in dairy cows and dairy calves. The key findings and mechanisms are summarized and potential further studies are suggested.

**Abstract:**

Calcium propionate is a safe and reliable food and feed additive. It can be metabolized and absorbed by humans and animals as a precursor for glucose synthesis. In addition, calcium propionate provides essential calcium to mammals. In the perinatal period of dairy cows, many cows cannot adjust to the tremendous metabolic, endocrine, and physiological changes, resulting in ketosis and fatty liver due to a negative energy balance (NEB) or milk fever induced by hypocalcemia. On hot weather days, cow feed (TMR or silage) is susceptible to mildew, which produces mycotoxins. These two issues are closely related to dairy health and performance. Perinatal period metabolic disease significantly reduces cow production and increases the elimination rate because it causes major glucose and calcium deficiencies. Feeding a diet contaminated with mycotoxin leads to rumen metabolic disorders, a reduced reproductive rate (increased abortion rate), an increased number of milk somatic cells, and decreased milk production, as well as an increased occurrence of mastitis and hoof disease. Propionic acid is the primary gluconeogenic precursor in dairy cows and one of the safest mold inhibitors. Therefore, calcium propionate, which can be hydrolyzed into propionic acid and Ca^2+^ in the rumen, may be a good feed additive for alleviating NEB and milk fever in the perinatal period of dairy cows. It can also be used to inhibit TMR or silage deterioration in hot weather and regulate rumen development in calves. This paper reviews the application of calcium propionate in dairy cows.

## 1. Introduction

There are many important challenges in dairy production, including reducing the feed intake and metabolic diseases caused by a negative energy balance (NEB) [1] and milk fever [2] during the perinatal period and mycotoxin pollution [3] of feed induced by environmental and climatic conditions, which have negative effects on milk production and quality and pose a potential threat to human health. In particular, ketosis and hypocalcemia represent two potentially devastating insults to the lactating dairy cow [4]. Appropriate dosages of calcium propionate as a feed additive can effectively alleviate these difficulties and serve the dairy industry.

Since calcium propionate does not inhibit yeast growth, it is one of the most useful antimicrobial preservatives in the fermented foods industry, especially in bread and fermented dairy products; in aqueous solution, it can dissociate to propionic acid (the active antifungal ingredient) and calcium ions [5]. It can be used as a feed preservative, growth promoter, intestinal microbiota enhancer, or appetite suppressant in animal nutrition [6]. In the dairy cow industry, calcium propionate can also be applied in many cases to inhibit mycotoxin production and as a metabolite precursor additive.

Propionic acid and Ca^2+^ are basic components in the rumen fluid [7], which means that calcium propionate is safe to add to the feed of dairy cows. It is approved by the World Health Organization (WHO) and the United Nations Food and Agriculture Organization (FAO) for use in food or feed additives. Therefore, it is used as a safe and valuable additive in the dairy cow industry. To update our knowledge on calcium propionate application for dairy cow performance and metabolism, we reviewed the effects of calcium propionate supplementation on decreasing feed mycotoxins, alleviating dairy cow NEB and milk fever, and promoting rumen development in dairy calves.

## 2. Properties of Calcium Propionate

### 2.1. Physical and Chemical Characteristics of Calcium Propionate

Calcium propionate is an organic salt formed by the reaction between calcium hydroxide and propionic acid [8] and has the molecular formula (CH_3_CH_2_COO)_2_Ca. The compound exists in either crystalline or powder form. Calcium propionate is soluble in water and can be hydrolyzed into Ca^2+^ and propionic acid. The solution is alkaline, but exerts bacteriostatic effects in acidic media. It is mainly synthesized through the reaction between a calcium source (CaCO_3_, CaO, Ca(OH)_2_, egg, or oyster shell) and propionic acid. Through the reaction, the generated calcium propionate is concentrated, dried, and dehydrated to obtain qualified products.

### 2.2. Antibacterial Properties of Calcium Propionate

Calcium propionate is a strong preservative with little or no flavor with normal use that can be effective against mold and bacteria and is widely used in foods, feeds, and pharmaceuticals [9]. It has the ability to inhibit the growth of molds and other microorganisms without an obvious inhibition of yeasts [8]. The antimicrobial properties of calcium propionate are dependent upon the corresponding undissociated acids in solution and involve the uncoupling of microbial substrate transport and oxidative phosphorylation from the electron transport system [10]. When exhibiting the same bacteriostatic effect, the effective dose of calcium propionate has been shown to be lower than that of sodium propionate. Calcium propionate has no teratogenic activity or reproductive toxicity, and propionic acid can be excreted in urine; thus, there is no risk of accumulation in the human body, even at large doses [5].

Propionic acid can interfere with the electrochemical gradients in the cell membrane, disrupt transport processes, and inhibit the uptake of substrate molecules, such as phosphate and amino acids [11]. The high-affinity potassium transporter in the cell membrane of yeasts enables the maintenance of pH homeostasis and stabilization of membrane potential by potassium uptake and accumulation, so the yeasts can develop tolerance to propionic acid [12]. However, the molds are susceptible to propionic acid. The antimicrobial activity of calcium propionate is due to the neutral undissociated propionic acid form, which is lipophilic and readily soluble in fungal cell membranes [5]. Therefore, the antimicrobial effect of calcium propionate depends on the pH value of the product because the undissociated acid has a better antimicrobial effect than the dissociated acid [13]. Compared to 71% undissociated propionic acid at pH 4.5, only 7% of the propionic acid can be undissociated at pH 6. An enhanced inhibitory effect of calcium propionate at a lower pH is expected, and the maximum pH at which calcium propionate exerts measurable antimicrobial activity is approximately 5.0–5.5 [13]. Therefore, a lower pH is beneficial to the improvement of the antibacterial properties of calcium propionate under appropriate conditions. Many antibacterial actions of calcium propionate have been found, and it can reduce the count of *Aspergillus flavus* [14], *Escherichia coli* O157:H7, *Salmonella enterica* serovar Typhimurium [15], Clostridia [16], and so on. Because of its antimicrobial properties, calcium propionate can also reduce mold and act as a preservative in many industries.

In the feed industry, a warm and humid climate and a long postharvest period favor mold growth and the production of mycotoxins [9]. Molds can cause economic losses and health problems due to the production of mycotoxins. Aflatoxins, which are potent mycotoxins mainly produced by *Aspergillus flavus* and *Aspergillus parasiticus*, have carcinogenic, mutagenic, teratogenic, and growth inhibiting effects on animals and humans [17]. Calcium propionate is a well-established chemical mold inhibitor that can be used in the feed industry to inhibit mold growth and reduce the incidence of aflatoxicosis in animals. Moreover, because of less feed spoilage, the heat production in feed is also reduced, preventing energy loss and poor palatability of the feed, which the cattle may refuse to eat. Bintvihok and Kositcharoenkul [14] indicated that aflatoxin B_1_–calcium propionate-supplemented diet groups showed increased body weight gain, feed consumption, and feed conversion, but decreased residual levels of aflatoxin B1 and aflatoxin M1 in muscle and liver tissues compared with those of aflatoxin B1-supplemented groups. Therefore, calcium propionate is a reliable additive for silage and TMR in dairy cows.

In other fields, calcium propionate has been used for paper preservation [5], increasing the fruit shelf life [18], and inhibiting bread spoilage [19], due to its antibacterial properties, and can also be used as an additive for silage and TMR in dairy cows.

### 2.3. Nutritive Properties of Calcium Propionate

As described above, calcium propionate can be hydrolyzed into Ca^2+^ and propionic acid. Calcium and propionic acid in solution are indispensable sources of nutrients for ruminants.

It is well-known that calcium is essential for the formation of skeletal tissue, transmission of nervous tissue, muscle contractility, and essential minerals for blood and milk. In contrast, the role of calcium with respect to the immune function and intermediary metabolism explains the contribution of subclinical hypocalcemia to the development of several diseases observed in early lactation and underlines its importance in high-performing dairy cows [20]. Oral calcium supplementation with calcium propionate, which is a calcium source in solution, can be greatly absorbed by the rumen and increase the ionized calcium concentration in the blood.

An increase in digestible energy intake has been shown to increase the posthepatic glucose supply [21]. Propionate, which is produced from the ruminal fermentation of starch and other organic matter, is the primary glucose precursor for ruminants [22]. Propionic acid, which is hydrolyzed from calcium propionate and formed under acidic conditions in the rumen, is absorbed by the rumen epithelium, passes to the liver through the portal vein, and synthesizes glucose. In fed cows, propionate is the major precursor of glucose, and the liver removal of propionate contributes to up to 60% of glucose hepatic release [23]. Propionate is an obligatory anaplerotic metabolite for the tricarboxylic acid (TCA) cycle [24]. It enters the TCA cycle through succinate, thus providing carbons that can either remain within the TCA cycle or be extracted from the cycle for gluconeogenesis [25]. Therefore, calcium propionate can be used as a good additive in ruminants for gluconeogenesis, and this property has been found to improve lamb performance [26], act as an energy source for finishing lambs [27], and improve beef quality [28]. Therfore, it is a favorable nutrient additive for dairy cows.

## 3. The Application of Calcium Propionate in Dairy Cows

As mentioned above, calcium propionate can be metabolized and absorbed by animals, providing them with essential calcium and glucose precursors, which are advantages that are not offered by other anti-mildew agents. Furthermore, it is generally regarded as safe (GRAS) in the United States, where upper limits only exist for its use in specific human food items [10]. Therefore, it is widely used in dairy cows as an antimicrobial agent, glucose precursor, and calcium provider.

### 3.1. Application in Silage to Resist Mildew

Silage is one of the most common ingredients in the diets of dairy cows and is an important source of nutrients. However, poorly made or contaminated silage can also be a source of pathogenic bacteria that may decrease the dairy cow performance, reduce the safety and quality of dairy products, and compromise animal and human health [29]. Molds identified in fermented feeds include *Aspergillus* sp., *Cladosporium* sp., *Fusarium* sp., *Mucor* sp., and *Penicillium* sp., and their adverse effects may occur through either their deleterious effects on the nutrient quality or their production of mycotoxins [30]. Several mycotoxins have been detected in corn silage, including aflatoxin B1, citrinin, deoxynivalenol, gliotoxin, and zearalenone [31].

To enhance the quality of silage, fermentation and the aerobic stability can be improved by adding silage additives. Propionic acid-based products, which are compatible with microbial inoculants, can be used as a silage additive. The combined use of propionic acid-based products and microbial inoculants can result in improvements in silage fermentation and the aerobic stability [32]. Propionic acid has excellent antifungal activity and has little impact on the activity of lactic acid bacteria. The application of propionic acid presents some problems due to its corrosive and hazardous nature, but its salt—calcium propionate—also has antimicrobial effects; additionally, it is safe and easy to handle [33]. Calcium propionate is an effective tool for suppressing the germination, growth rate, and aflatoxin production of *Aspergillus flavus* (A-2092) in different substrates [34]. Therefore, calcium propionate has the potential, as an additive in silage, to inhibit the growth of molds and decrease the mycotoxin contents in silage.

Alfalfa is a protein-rich forage that is widely cultivated and has become a major protein source of diets for dairy cows. Fresh alfalfa silage is prone to clostridia spoilage because of its low dry matter, low sugar contents, and high buffering capacity [33]. Dong et al. [33] demonstrated that the amount of enterobacteria, molds, and clostridia decreased linearly with an increasing calcium propionate proportion in alfalfa silage, while lactic acid bacteria counts quadratically increased; calcium propionate can improve the fermentation quality and aerobic stability of proteolysis alfalfa silage, and the recommended additive level is 10 g/kg fresh weight. Wen et al. [16] evaluated the potential of calcium propionate as an alfalfa silage additive and found that calcium propionate decreased the butyric acid content and dry matter loss and increased the water-soluble carbohydrate content. However, the pH decreased slowly at the start of ensilage, possibly because of the alkaline properties of calcium propionate. After 30 days of storage, calcium propionate increased the concentrations of lactic, acetic, propionic, and total organic acids and the microbial populations of lactic acid bacteria, but decreased the enterobacteria, mold, and clostridia populations [35]. The lower population of molds and clostridia may be related to the antimicrobial effects of calcium propionate. Clostridium is a kind of undesirable microorganism that is harmful to animal health. It not only destroys lactic acid, but also leads to an increase in the pH value and a decrease in the nutritional value of silage [16]. In conclusion, calcium propionate is a good additive for silage, which can act as a significant inhibitor for the growth of molds and clostridia.

### 3.2. Application in TMR to Increase the Aerobic Stability

Warm and humid conditions are favorable for mold growth and can result in increased mycotoxin production. The spoilage of TMR in summer is an important factor affecting the production efficiency. To reduce the influence of TMR mold growth and its metabolites on the production performance, health, and milk quality of cows, appropriate methods, including chemical additives, water content control, increasing the number of fresh feed deliveries per day, and the timely cleaning of leftovers, must be used. As mentioned above, calcium propionate is a safe and effective inhibitor of mold, and can improve the aerobic stability of feed. Mold growth can be prevented in coarse texture feeds and other high moisture feeds by the addition of calcium propionate [14]. The addition of calcium propionate to TMR inhibits feed spoilage. Therefore, the proper addition of calcium propionate in TMR feed may have the function of preventing feed corruption. However, research on the recommended amount of calcium propionate added to TMR to prevent feed spoilage is needs to be further explored.

### 3.3. Application as a Gluconeogenic Precursor to Alleviate NEB in the Perinatal Period

The perinatal period from late pregnancy to early lactation is a critical period in a dairy cow’s life due to the rapidly increased drain of nutrients from the mother towards the fetus and into the colostrum and milk [36]. After calving, with a high yield of milk, the nutrient intake of dairy cows is large under the need to supply the output of milk, resulting in a negative nutrient balance that requires the mobilization of body reserves. The metabolic diseases fatty liver and ketosis are due to the extent of glucose deficit that induces the excessive mobilization of body fat. In addition, during the perinatal period, the dry matter intake of dairy cows is reduced due to the diminution in rumen volume induced by the growth of the fetus and other hormonal changes [37]. The high energy demands of lactation, coupled with a reduction in the dry matter intake around calving, means that the majority of dairy cows enter the state of NEB in early lactation [38]. Cows showing excessive NEB utilize their body fat as a source of energy to maintain the rapidly increasing milk yield, which leads to excessive body fat mobilization, ketosis, and fatty liver syndrome. Metabolic or infectious diseases, including fatty liver syndrome and ketosis, affect dairy cow production during the perinatal period [39] and further impact the welfare, productive lifespan, and economic outcomes of dairy cows [36].

Strategies for supplying energy are one way of mitigating NEB. Based on a large amount of data on cattle and other species, glucose is known to reduce the fatty acids mobilized from adipose tissue [40]. The failure of cows to meet their glucose demands for lactation leads to an impaired immune response and an increased risk of disease that may affect milk production and profitability [41]. For cow rearing, the dietary energy can be improved through fat or concentrate supplementation to alleviate NEB, but excess fat supplementation inhibits rumen microbial growth, decreases the rumen pH value, and increases the rate of subclinical ruminal acidosis [42].

Glucose precursors, such as propylene glycol and calcium propionate, have been used in dairy cattle to correct metabolic problems [43]. Propionate can directly regulate its own metabolism in isolated bovine hepatocytes through upregulation of the mRNA expression of cytosolic phosphoenolpyruvate carboxykinase (PCK1), mitochondrial phosphoenolpyruvate carboxykinase (PCK2), and pyruvate carboxylase (PC), which are the key enzymes required for the stimulation of gluconeogenesis from propionate in ruminants [44]. Propionate is the major glucose precursor in ruminants that has a positive energy balance and anti-ketogenic effects [45]. It is used as a readily available energy source to correct metabolic problems in dairy cattle [46]. Propionate, whose liver uptake is preferential and highly efficient, can inhibit hepatic lipid oxidation and the production of ketones [47].

During the perinatal period, calcium propionate is a good available energy source for preventing metabolic disorders in dairy cows, so it can be incorporated into the diet and increase the rumen concentration of propionate, which is the main precursor for glucose synthesis in the liver [27]. Abdel-Latif et al. [39] found that the supplementation of calcium propionate in primiparous Egyptian buffalo cows during late gestation and early lactation significantly improved the body weight, reproductive parameters such as first estrus postpartum, days open, and number of services per conception; it also significantly decreased the blood metabolites of nonesterified fatty acids (NEFAs) and increased the glucose and insulin concentrations. The beta-hydroxybutyrate (BHBA) can be considered an indicator of a negative energetic balance due to its correlation with the energetic demand and energy reserves [48]. In the study of Martins et al. [48], the calcium propionate-supplemented group had a lower amount of blood BHBA because the propionate is the principal source of gluconeogenesis in peripartum cows. Maintaining or increasing the DM intake is also crucial for alleviating NEB in the perinatal period. The effects of calcium propionate on the DM intake in dairy cows are controversial; some studies have reported no difference [46], and some have reported a higher DM intake with calcium propionate [40]. McNamara and Valdez [40] showed that cows fed 0.125 kg/d calcium propionate increased their DM intake by 11% and 13% in the prepartum and postpartum groups, respectively, compared with that of the control group, and showed reduced net lipolysis, but increased adipose tissue lipogenesis, at postpartum. Martins et al. [49] confirmed that, during early lactation, 200 g/d calcium propionate provided a better energetic supply for dairy cows, which can also increase the milk yield and protein, lactose, fat, and total solids contents in milk, despite the reduced dry matter intake. Propionate is converted to glucose in the liver, supporting lactose synthesis in the mammary gland [49]. Liu et al. [46] observed that increasing the supplementation of calcium propionate improved the energy status, as indicated by the higher blood glucose, lower blood BHBA and NEFA, and lower urine ketones. In ruminants, an increase in glucose precursors, such as propionate, could optimize nutrient use and improve milk production [50]. The concentration of oxaloacetate determines whether acetyl-CoA enters the TCA-cycle or ketogenesis occurs. The antiketogenic effect of calcium propionate is also related to the increase of the oxaloacetate content in the mitochondria of the liver. Calcium propionate can be incorporated into the diet or fed per cow per day for cows in early lactation. The optimum dose was approximately 200 g per cow per day in the experimental conditions of Liu et al. [46]. In conclusion, calcium propionate can act as a good glucose precursor to alleviate NEB in dairy cows. However, the maximum dose available in cows still needs to be studied because calcium propionate can depress appetite [6].

### 3.4. Application as a Source of Calcium to Prevent Milk Fever in the Perinatal Period

Milk fever is a metabolic disease characterized by clinical symptoms due to a reduction in the blood calcium concentration (hypocalcemia) during peripartum, which affects high-yielding multiparous cows [51]. It is one of the most common periparturient abnormalities afflicting dairy cows [52]. Milk fever can decrease the dry matter intake, milk production, and reproductive performance and increase the risk of secondary diseases, such as ketosis, a retained placenta, displaced abomasum, mastitis, and the incidence of dystocia and uterine disorders [53,54]. When the concentration of blood calcium falls below a critical threshold, it results in clinical and subclinical milk fever [54,55]. Serum calcium levels of 2 and 1.4 mmol/L have been proposed as thresholds of subclinical and clinical hypocalcemia, respectively, but the external signs may not be displayed in dairy cows [56]. Improving the mobilization of calcium from bone and the absorption of calcium from the diet are two major processes that prevent the decrease in blood calcium in dairy cows. The mobilization of calcium from bone can be accomplished by feeding a calcium-deficient diet or negative dietary cation-anion difference in the pre-calving period [57]. In addition, the infusion of 5-hydroxytryptophan can also improve blood calcium concentrations around parturition [58].

However, after calving, it is important to improve the available calcium in the diet for absorption. It is well-accepted that calcium can be absorbed across the rumen wall of sheep and goats if the soluble calcium concentration is high [59,60]. Schroder et al. [61] proved that calcium can be absorbed across the cattle rumen epithelium in vitro. Calcium absorption by the rumen appears to be a key factor in calcium homeostasis at the onset of lactation, and its failure causes uncontrolled hypocalcemia, leading to parturient paresis [62]. Calcium sources that are soluble at a slightly acidic pH may result in more absorption from the rumen, intestine, or both, than insoluble calcium sources [63]. To increase the calcium absorption, an effective method is to increase the concentration of ionized calcium within the rumen by the given supplement [61]. Providing a highly soluble source of oral calcium induces high concentrations of ionized calcium in the lumen of the gastrointestinal tract. The high concentrations of ionized calcium in the rumen lumen induce a chemical gradient that passively transports ionized calcium from the mucosa through the tight junctions towards the extracellular space on the serosa side, increasing the concentrations of ionized calcium in the blood [64]. The administration of a ruminal calcium bolus (approximately 43 g of calcium) has been used to restore blood calcium concentrations [57] by improving the amount of calcium that can be absorbed from feed.

Calcium chloride solutions and gel products offer good calcium sources that are very soluble, very concentrated (36% calcium), and rapidly absorbed, making them generally effective in reducing the incidence of milk fever [65]. Large amounts of propionic acid are produced in the rumen by carbohydrate metabolism, and there are no obvious adverse effects, so calcium propionate might also be a satisfactory source of calcium [66]. Although its effects on blood calcium are not as rapid as those of calcium chloride, calcium propionate can be used in the form of calcium propionate paste and given at calving or after calving in dairy cows to prevent milk fever. Pehrson et al. [66] showed that the incidence of milk fever in a calcium propionate-treated (120 g of calcium in total) group of cows that experienced milk fever during previous calving was 25.3%, which was lower than that in untreated cows (36.0%), but similar to that in the calcium chloride-treated group (23.2%). Therefore, calcium propionate is considered a satisfactory alternative to calcium chloride for the prevention of milk fever [66]. Goff et al. [65] also demonstrated that calcium propionate paste treatment was beneficial in reducing subclinical hypocalcemia and could reduce the incidence of milk fever from 50% in control cows to 29% in treated cows. Calcium propionate is less soluble in water than calcium chloride, but its solubility is adequate and more soluble than that of calcium lactate, calcium sulfate, and calcium carbonate. Calcium propionate is neutral in taste and has no erosive effect on the digestive tract mucosa [66]. To prevent milk fever, calcium propionate is usually administered orally around the time of the calving of cows. Kara et al. [67] found that giving cows two drenches (each drench contained 0.68 kg calcium propionate) at calving and 24 h after calving was beneficial in treating milk fever. In summary, calcium propionate can act as a source of calcium to prevent milk fever in dairy cows during the perinatal period.

### 3.5. Application in Dairy Calves to Regulate Rumen Development or Improve Growth

The rumen is a vital digestive organ that plays a key role in the growth, production performance, and health of ruminants. Therefore, promoting rumen development has always been a key target of calf nutrition [68]. The papilla length of the rumen is the most important factor for the evaluation of rumen development [68]. Rumen epithelium development plays a very important role in the absorption, metabolism, and transportation of volatile fatty acids (VFAs). VFAs, such as propionic and butyric, provide the main chemical stimuli for the proliferation of the rumen epithelium if the amount is sufficient [69], indicating that additives of propionate may be used in calf feed as rumen growth promoters. As one kind of propionate, the additive of calcium propionate may also stimulate the epithelium development of calves. G protein-coupled receptors (GPRs) are integral membrane proteins which are activated by an external signal in the form of a ligand or other signal mediator [7]. Zhang et al. [7] found that calves supplemented with 5% calcium propionate (mixed in milk replacer and starter ration) in the diet had a greater rumen papillae length and improved mRNA expression of G protein-coupled receptor 41 (GPR41), GPR43, and cyclin D1 after feeding for 160 days, which indicated that propionate acted as a signaling molecule to improve the rumen epithelium. Propionate can be converted into glucose in the liver, and higher glucose concentrations mean that high energy can be used to increase the body weight of calves. Zhang et al. [70] pointed out that there were no differences in DMI with the different feeding levels of calcium propionate, but the addition of calcium propionate improved the growth performance and gastrointestinal tract traits of Jersey calves; thus, adding 10% calcium propionate to the feed before 90 days and 5% for 90 to 160 days was beneficial for calves. Cao et al. [71] also verified that calcium propionate supplementation (5% dry matter) can improve body weight gain and rumen growth both pre- and postweaning. Monensin is an ion carrier that can change the number of rumen microorganisms, reduce the amount of methane production, increase propionate in the rumen, decrease the intake of dry matter, and improve the efficiency of milk production and weight gain of dairy cows [72]. However, as an antibiotic, the use of monensin in animal feed as a growth promoter may enhance the risk of antibiotic-resistant strains, so it is important to seek alternatives to this compound [73]. Ferrerra and Bittar [74] revealed that employing calcium propionate as an additive in starter feeds of calves resulted in an equal animal performance before and after weaning in comparison to that of sodium monensin, which suggests that sodium monensin may be replaced by calcium propionate. Therefore, calcium propionate can be used as a good additive to promote the rumen development and growth of dairy calves.

## 4. Limitation of Calcium Propionate in Application

However, the use of calcium propionate in dairy cows should be controlled at appropriate doses because an overdose has a hypophagic effect in ruminants [27] and may decrease the DMI of dairy cows. It has also been reported that calcium propionate induces a negative causation state while reducing the feed intake in broiler breeders [6], rats [75], and steers [63] at high doses. The metabolic feedback theory contends that when the absorption of nutrients, principally energy and protein, exceeds the requirements, negative metabolic feedback impacts DMI. Calcium propionate is an important energy provider when working as an additive to alleviate NEB in dairy cows. Therefore, propionic acid is the fuel most likely to stimulate satiety and reduce the feed intake in dairy cows [76] because it has a high energy concentration. Propionic acid can stimulate the oxidation of acetyl CoA in the liver [24]. According to the oxidation theory, the oxidation of fuels in the liver can stimulate satiety by transmitting signals via hepatic vagal afferents to feeding centers in the brain [77]. Oba and Allen [78] confirmed that a propionate infusion linearly decreased the DMI of dairy cows at higher doses. When feeding calcium propionate at a high level, the TCA cycle intermediates increase, stimulating the oxidation of acetyl CoA, likely affecting the feeding behavior and satisfaction. However, propionate had a smaller hypophagic effect at low plasma glucose concentrations and had a greater hypophagic effect at elevated plasma glucose concentrations [79]. Therefore, when appetite reduction occurs in cows, the supply of calcium propionate suppresses the requirement for gluconeogenesis. However, the maximum dose available in cows remains to be determined.

## 5. Conclusions

**Figure 1:**
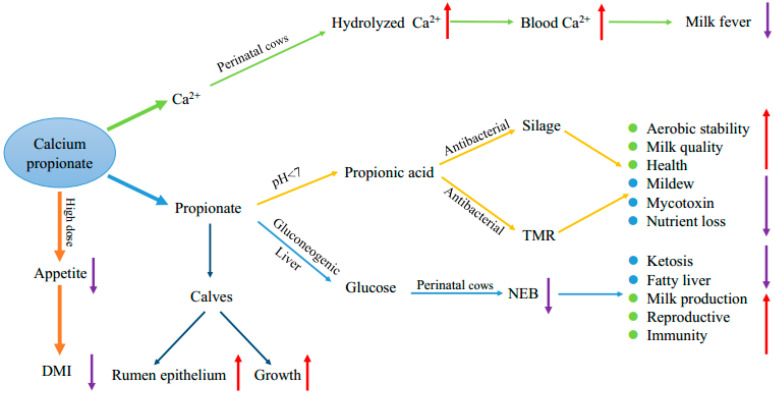
Summary of the calcium propionate applications in dairy cows. The effects of supplementation with calcium propionate in dairy cows can be divided into the functions of hydrolyzed Ca^2+^ and propionic acid. Ca^2+^, as a water-soluble calcium source, can reduce the incidence of milk fever by increasing the calcium content in the blood of perinatal dairy cows. Propionic acid is mainly used to inhibit the growth of mildew in silage or total mixed rations (TMR) or as a glucose precursor to inhibit a negative energy balance (NEB). Propionate can also promote the development of the rumen epithelium of calves. However, at very high doses, calcium propionate may lead to a decrease in appetite.

Calcium propionate is a safe and reliable food and feed additive. The calcium ions and propionic acid generated by calcium propionate hydrolysis are the basic components in the rumen of dairy cows. The application of calcium propionate in dairy cows is summarized in Figure 1. Calcium propionate can be used as a silage additive to inhibit the growth of mildew, reduce mycotoxins, and improve the aerobic stability. Adding calcium propionate to TMR feed can inhibit the putrefaction of the feed in hot weather; thus, calcium propionate can also be used as a preservative in feed. Propionic acid generated by the hydrolysis of calcium propionate is the main glucose precursor in dairy cows. Adding calcium propionate to the diet during the perinatal period can effectively alleviate the nutritional metabolic disease caused by NEB in dairy cows, and the calcium ions generated by hydrolysis can serve as a calcium source to effectively alleviate paralysis caused by milk fever. Calcium propionate can also promote dairy calves’ rumen epithelium development to promote calf growth through propionic acid. Proper feeding is beneficial to the health of dairy cows, but excessive feeding may inhibit the appetite and limit the intake. In summary, the applications of calcium propionate in dairy cows mainly include the inhibition of feed mildew, alleviation of NEB, prevention of milk fever, and promotion of the rumen epithelial development of dairy calves.

The following are some of the limitations of the current research and areas to be explored:

(1) Currently, research on calcium propionate in dairy cows has been mainly carried out by oral feeding alone, which is not convenient for application in practical production. Therefore, to improve the application effectiveness of calcium propionate in dairy cows, more studies are needed to determine the optimal feeding level when calcium propionate is mixed with TMR. When calcium propionate is used to prevent NEB and milk fever, the optimal feeding ratio should be revealed according to a cows’ milk production level and body condition;
(2) Excessive calcium propionate feeding has been shown to inhibit the appetite and limit the intake. To avoid its adverse effects, the maximum feeding level, influencing factor, and adverse impact of calcium propionate in dairy cows need to be further studied;
(3) Calcium propionate can be used as an anti-mildew additive in silage and can also be directly added to feed to prevent several metabolic diseases of dairy cows during the perinatal period. However, few studies on the effects of silage with calcium propionate on perinatal dairy cows have been conducted. Therefore, research in this field is worth exploring.
